# USP15 targets ALK3/BMPR1A for deubiquitylation to enhance bone morphogenetic protein signalling

**DOI:** 10.1098/rsob.140065

**Published:** 2014-05-21

**Authors:** Lina Herhaus, Mazin A. Al-Salihi, Kevin S. Dingwell, Timothy D. Cummins, Lize Wasmus, Janis Vogt, Richard Ewan, David Bruce, Thomas Macartney, Simone Weidlich, James C. Smith, Gopal P. Sapkota

**Affiliations:** 1 Medical Research Council Protein Phosphorylation and Ubiquitylation Unit, University of Dundee, Dow St., Dundee DD1 5EH, UK; 2 Division of Systems Biology, MRC National Institute for Medical Research, The Ridgeway, Mill Hill NW7 1AA, UK

**Keywords:** bone morphogenetic protein, deubiquitylation, USP15, ubiquitin, ALK3, SMAD

## Abstract

Protein kinase ALK3/BMPR1A mediates bone morphogenetic protein (BMP) signalling through phosphorylation and activation of SMADs 1/5/8. SMAD6, a transcriptional target of BMP, negatively regulates the BMP pathway by recruiting E3 ubiquitin ligases and targeting ALK3 for ubiquitin-mediated degradation. Here, we identify a deubiquitylating enzyme USP15 as an interactor of SMAD6 and ALK3. We show that USP15 enhances BMP-induced phosphorylation of SMAD1 by interacting with and deubiquitylating ALK3. *RNAi*-mediated depletion of USP15 increases ALK3 K48-linked polyubiquitylation, and reduces both BMP-induced SMAD1 phosphorylation and transcription of BMP target genes. We also show that loss of USP15 expression from mouse myoblast cells inhibits BMP-induced osteoblast differentiation. Furthermore, USP15 modulates BMP-induced phosphorylation of SMAD1 and transcription during *Xenopus* embryogenesis.

## 2. Introduction

Bone morphogenetic proteins (BMPs) are members of the transforming growth factor beta (TGFβ) family of cytokines. BMPs play crucial roles in embryogenesis and tissue homeostasis. Aberrant BMP signalling is associated with developmental defects as well as several human diseases [1–7]. BMPs signal through phosphorylation and activation of type 1 BMP receptor kinases, including ALK3/BMPR1A, which then phosphorylate intracellular SMAD transcription factors 1, 5 and 8 at their C-terminal SXS motif [8,9]. Phosphorylation of SMADs 1/5/8 induces their interaction with SMAD4 and translocation to the nucleus, where along with other cofactors they regulate transcription [8,9]. Context-specific transcriptional programmes controlled by BMP signalling are central to the regulation of cell differentiation, proliferation, apoptosis and migration [10–12]. The action of BMPs in cells and tissues is therefore tightly regulated at multiple steps of the BMP pathway. Together, a wide range of regulators shape the action of BMP through key biological processes including the establishment of the body axis and neuralization of the early embryo as well as osteogenesis and bone formation in adults [2–4,11,13].

Reversible ubiquitylation of multiple BMP pathway components is one of the key mechanisms to dynamically fine-tune BMP signalling [14]. The inhibitory SMADs 6 and 7, which are transcriptional targets of BMP signalling, are part of a negative feedback loop regulating the pathway [14–17]. SMAD6 selectively binds type I BMP receptor ALK3, thereby limiting its ability to associate with and phosphorylate SMADs 1, 5 and 8 [18]. SMAD6 also recruits the E3 ubiquitin ligases SMURF1/2 to type I BMP receptors, targeting them for ubiquitin-mediated degradation [15,19].

Polyubiquitin chains attached to target proteins can be edited or removed by deubiquitylating enzymes (DUBs), adding a further layer of control to signalling. DUBs that target and remove ubiquitin chains from type I BMP receptors have not yet been identified. However, several DUBs have been implicated in the control of type I TGFβ receptors. For example, the closely related DUBs USP4, USP11 and USP15 have been reported to modulate TGFβ signalling by deubiquitylating the type I TGFβ receptor ALK5 [20–22]. USP15 has also been reported to act on monoubiquitylated R-SMADs [23].

In the course of a proteomic approach to identify novel regulators of the BMP pathway, we identified USP11 and USP15 as SMAD6 interactors. Here, we demonstrate that USP15 interacts with and deubiquitylates the type I BMP receptor ALK3. Further work reveals USP15 as a key player in the BMP pathway in human and mouse cells as well as *Xenopus* embryos, influencing BMP-dependent SMAD1 phosphorylation, gene transcription and osteoblastic differentiation.

## 3. Results

### 3.1. Identification of USP15 as an interactor of SMAD6

**Figure 1.**
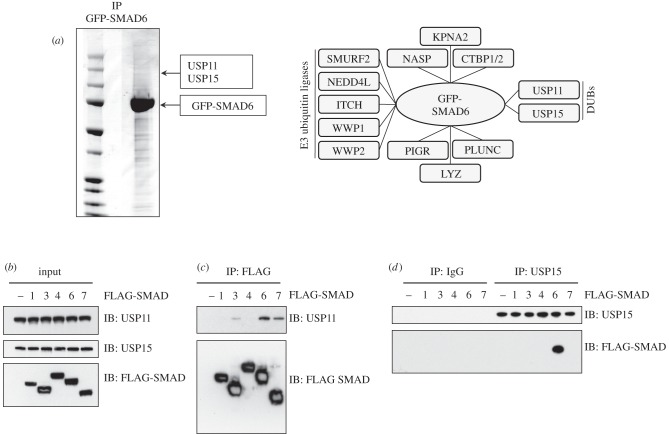
Identification and characterization of USP15 as an interactor of SMAD6. (*a*) Coomassie stained gel image showing anti-GFP-IPs from HEK293 extracts expressing GFP-SMAD6. The interacting proteins were excised as 2 mm gel pieces, digested with trypsin and identified by mass spectrometry. The location where USP11 and USP15 were identified is indicated. All protein interactors of GFP-SMAD6 identified by mass spectrometry are indicated in the right panel. Protein interactors of GFP control were removed from the list of GFP-SMAD6 interactors. (*b*) HEK293 cells were transfected transiently with FLAG-SMADs. Extract inputs were resolved by SDS-PAGE and subjected to immunoblotting (IB) with anti-USP11, anti-USP15 and anti-FLAG antibodies as indicated. (*c*) FLAG-IPs were subjected to immunoblotting with anti-USP11 and anti-FLAG antibodies. (*d*) Endogenous pre-immune IgG or anti-USP15 IPs were subjected to immunoblotting with anti-FLAG and anti-USP15 antibodies as indicated.

In an effort to uncover new regulators of the BMP pathway, we employed a proteomic approach to identify interactors of SMAD6. We stably integrated a single copy of GFP-tagged SMAD6 into human embryonic kidney (HEK293) cells under a tetracycline-inducible promoter. Treatment of these cells with tetracycline resulted in a robust expression of GFP-SMAD6. Immunoprecipitates (IPs) of GFP-SMAD6 from cell extracts were resolved by SDS-PAGE and the interacting proteins were excised, digested with trypsin and identified by mass spectrometry. Consistent with the reported roles of SMAD6 in recruiting E3 ubiquitin ligases to ALK3 [14,17,19,24], we identified several members of the HECT E3 ubiquitin ligase family, including SMURF2, WWP1/2, NEDD4L and ITCH, as interactors of GFP-SMAD6 (figure 1*a*). Interestingly, two DUBs (USP11 and USP15) were identified as novel interactors of GFP-SMAD6 (figure 1*a*). USP11 and USP15 did not feature as interactors of either GFP alone or of GFP-tagged SMADs 1–5 and 8 in similar proteomic assays [20,25]. Several other proteins, including NASP, CTBP1/2, KPNA2, PIGR, PLUNC and LYZ were also identified as interactors of only GFP-SMAD6 (figure 1*a*).

We first investigated the specificity of interactions of SMAD6 with USP11 and USP15. To this end, an empty vector control or FLAG-tagged SMADs 1, 3, 4, 6 and 7 were transiently transfected into HEK293 cells (figure 1*b*). Endogenous USP11 was detected predominantly in FLAG-SMAD6 and FLAG-SMAD7 IPs compared with IPs of other FLAG-SMADs (figure 1*c*). When co-expressed in HEK293 cells, both FLAG-SMAD6 and FLAG-SMAD7 IPs pulled down HA-USP11 to a similar extent (electronic supplementary material, figure S1). This confirms our previous observation that USP11 interacts with SMAD7 [20].

To detect interactions between USP15 and FLAG-tagged SMADs, endogenous USP15 was immunoprecipitated from HEK293 cell extracts transfected with either empty vector control or FLAG-tagged SMADs 1, 3, 4, 6 and 7 (figure 1*b*). FLAG-SMAD6, but none of the other FLAG-SMADs, was detected in USP15 IPs (figure 1*d*), indicating the selective nature of the interaction between SMAD6 and USP15. This is consistent with our previous observations showing that even under overexpression conditions, HA-USP15 does not interact with FLAG-SMADs 1, 3, 4 and 7 [20].

Analysis of the expression of USP11 and USP15 in mouse tissues showed that USP15 is expressed ubiquitously, whereas USP11 was restricted to the brain, spleen, thymus and pancreas, with almost no observable expression elsewhere (electronic supplementary material, figure S2).

### 3.2. Depletion of USP15 inhibits bone morphogenetic protein pathway signalling

SMAD6 inhibits BMP signalling, in part, by recruiting E3 ubiquitin ligases to BMP receptors and targeting them for ubiquitin-mediated degradation [14,15,18,19]. The association of USP11 and USP15 with SMAD6 implied a possible role for these DUBs in the BMP pathway. We have previously reported that USP11, which interacts with both SMAD6 and SMAD7, enhances TGFβ signalling through ALK5 deubiquitylation [20]. The selective nature of the interaction between USP15 and SMAD6 prompted us to investigate a possible role for USP15 in BMP signalling.

**Figure 2.**
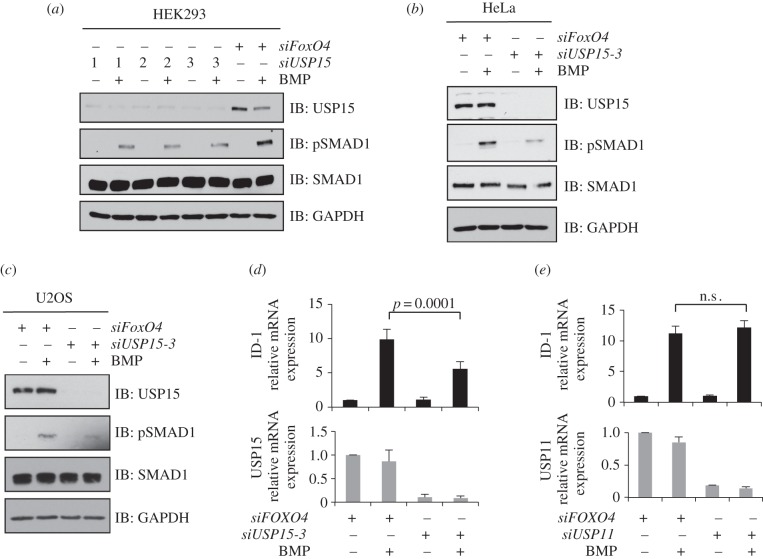
Depletion of USP15 inhibits BMP signalling. (*a*) HEK293 cells were transiently transfected with three individual *siRNAs* targeting USP15, serum-starved overnight and stimulated with 6.25 ng ml^−1^ BMP for 1 h prior to lysis. Extracts were resolved by SDS-PAGE and subjected to immunoblotting with antibodies against endogenous USP15, pSMAD1, SMAD1 and GAPDH. (*b*) As in (*a*), except that *siUSP15-3* was used to knockdown endogenous USP15 expression in HeLa cells. (*c*) As in (*b*) except that U2OS cells were used. (*d*) HEK293 cells were transiently transfected with *siUSP15-3.* Cells were serum-starved overnight and stimulated with 6.25 ng ml^−1^ BMP for 1 h. Cells were then washed and harvested 2 h later. The expression of USP15 and the BMP-target gene ID1 were assessed by qRT-PCR. Results are average of six biological replicates. The error bars indicate s.d. (*e*) As in (*d*), except that HEK293 cells were transfected with *siUSP11.* The expression of USP11 and ID1 were assessed by qRT-PCR. Results are average of three biological replicates. The error bars indicate s.d.

We therefore investigated the effect of *RNAi*-mediated depletion of USP15 in BMP signalling in three different human cell lines. Three distinct *siRNAs* targeting USP15 and a control *siRNA* targeting FoxO4 were transfected in HEK293 cells (figure 2*a*), in which all three USP15 *siRNAs* caused an approximately 80–90% reduction in USP15 protein levels compared with the FoxO4 control (figure 2*a*). Depletion of USP15 caused a substantial reduction in the levels of BMP-induced pSMAD1 (tail-phosphorylated SMAD1) without significantly affecting total SMAD1 levels (figure 2*a*). The inhibition of BMP-induced pSMAD1 levels by *siUSP15-3* was partially rescued by the restoration of FLAG-USP15 overexpression in cells (electronic supplementary material, figure S4).

In HeLa cervical cancer cells (figure 2*b*) and U2OS osteosarcoma cells (figure 2*c*), transfection of *siUSP15-3* caused an almost complete loss of endogenous USP15 protein expression. This caused a significant reduction in the levels of BMP-induced pSMAD1, while the total SMAD1 levels were not altered compared with *siFoxO4* control (figure 2*b,c*).

We also investigated the effects of USP15 depletion on BMP transcriptional activity in HEK293 cells. BMP induces expression of the inhibitor of differentiation 1 (ID1) gene [26]. *RNAi*-mediated depletion of USP15 in HEK293 cells reduced the expression of ID1 mRNA significantly in response to BMP treatment compared with *siFoxO4* control (figure 2*d*). By contrast, depletion of USP11, which inhibits TGFβ-dependent transcription [20], did not inhibit BMP-induced expression of ID1 mRNA (figure 2*e*). Together our results suggest that USP15 is critical for BMP-induced phosphorylation of SMAD1 and downstream transcriptional activity, whereas USP11 is unlikely to affect BMP signalling. Consistent with the reported role for USP15 in TGFβ-dependent transcription [21], we also found that depletion of USP15 from TGFβ-treated HaCaT cells resulted in a reduction of plasminogen activator inhibitor 1 (PAI1) expression compared with *siFoxO4* control (electronic supplementary material, figure S3).

### 3.3. USP15 enhances bone morphogenetic protein pathway signalling, and interacts and co-localizes with ALK3

**Figure 3.**
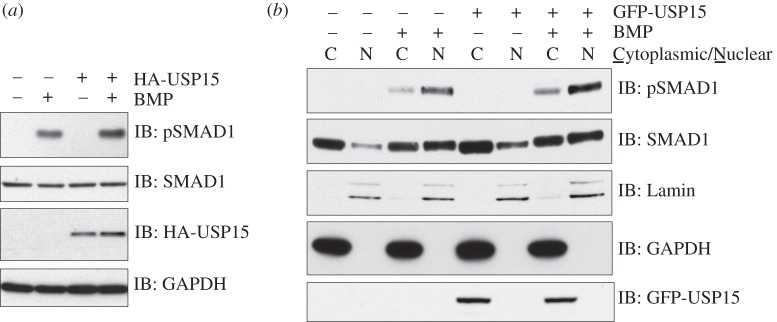
USP15 augments BMP signalling. (*a*) HEK293 cells transiently expressing control HA-vector or HA-USP15 were serum-starved overnight and stimulated with 6.25 ng ml^−1^ BMP-2 for 1 h prior to lysis. Extracts were resolved by SDS-PAGE and subjected to immunoblotting with antibodies against HA, endogenous pSMAD1, total SMAD1 and GAPDH. (*b*) HEK293 cells stably expressing GFP or GFP-USP15 were serum-starved overnight and stimulated with 6.25 ng ml^−1^ BMP for 1 h prior to separation into cytoplasmic and nuclear fractions. The fractions were resolved by SDS-PAGE and subjected to immunoblotting with antibodies against GFP, Lamin A/C, GAPDH, endogenous pSMAD1 and total SMAD1.

As depletion of USP15 inhibits BMP signalling, we asked whether elevation of USP15 has the opposite effect. Indeed, overexpression of HA-USP15 in HEK293 cells increased the levels of pSMAD1 in response to BMP signalling (figure 3*a*), and this was true in both nuclear [27–29] and cytoplasmic fractions (figure 3*b*). Cytoplasmic fractions also contained the majority of GFP-USP15 (figure 3*b*).

The enhanced BMP signalling due to USP15 overexpression suggests that SMAD6 itself is unlikely to be a substrate of USP15; deubiquitylation and stabilization of SMAD6 by USP15 would be expected to inhibit BMP signalling. The observation that SMAD1 levels are unaffected by either USP15 overexpression or depletion suggests that the target of USP15 in the BMP pathway is upstream of SMAD1. The type I BMP receptor ALK3 lies immediately upstream of SMAD1 in the BMP pathway, and ALK3 is targeted for ubiquitylation by SMAD6 via recruitment of E3 ubiquitin ligases [19]. We hypothesized that USP15 deubiquitylates ALK3, thereby opposing the effect of SMAD6 and its associated E3 ubiquitin ligases.

**Figure 4.**
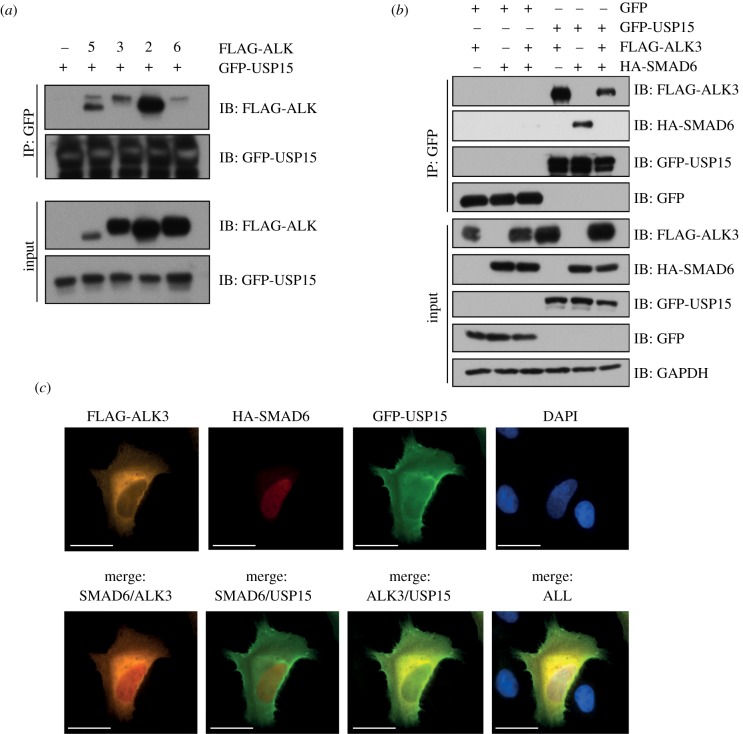
USP15 interacts and co-localizes with SMAD6 and ALK3. (*a*) HEK293 cells were transfected with GFP-USP15 with control vector or mammalian expression vectors encoding N-terminal FLAG-tagged ALK5, ALK3, ALK2 or ALK6. Cells were lysed and extracts (1 mg) subjected to GFP-IPs. GFP-IPs (40%) or extract inputs were resolved by SDS-PAGE and subjected to immunoblotting with the indicated antibodies. (*b*) HEK293 cells expressing GFP control or GFP-USP15 were transfected with FLAG-ALK3, HA-SMAD6 or both as indicated. Cells were lysed and extracts (1 mg) subjected to GFP-IPs. GFP-IPs (40%) or extract inputs were resolved by SDS-PAGE and subjected to immunoblotting with the indicated antibodies. (*c*) Fixed cell immunofluorescence was performed on U2OS cells transfected with FLAG-ALK3, HA-SMAD6 and GFP-USP15. Individual and merged pictures are shown, indicating localization of FLAG-ALK3 mainly in the cytosol, HA-SMAD6 in the nucleus and GFP-USP15 in both compartments. GFP-USP15 mainly co-localizes with FLAG-ALK3. Pictures were taken using a 60× lens, scale bar represents 30 μm.

To explore this idea, we first tested the ability of USP15 to interact with various ALKs, including ALK3, upon co-expression in HEK293 cells. GFP-USP15 IPs from HEK293 extracts interacted with FLAG-ALK5, FLAG-ALK3, FLAG-ALK2 and FLAG-ALK6 (figure 4*a*). Under these conditions, the association between USP15 and ALK2 appeared to be the strongest (figure 4*a*). Next, we tested how SMAD6 affected the interaction between GFP-USP15 and FLAG-ALK3 (figure 4*b*). Expression of SMAD6 caused a reduction in the ability of GFP-USP15 to interact with FLAG-ALK3, suggesting that SMAD6 disrupts USP15 : ALK3 association. Interestingly, the interaction between GFP-USP15 and HA-SMAD6 was completely abolished by FLAG-ALK3 overexpression (figure 4*b*), suggesting that USP15 : SMAD6 and USP15 : ALK3 interactions could be mutually exclusive.

In order to probe this possibility, we employed immunofluorescence studies in U2OS osteosarcoma cells. In the absence of SMAD6 or ALK3 overexpression, GFP-USP15 expression in U2OS cells was observed to be pan-cellular (electronic supplementary material, figure S5a). When HA-SMAD6 was co-expressed with GFP-USP15, SMAD6 was observed mainly in the nucleus but also in the cytoplasm (electronic supplementary material, figure S5b). There was a significant overlap in expression between GFP-USP15 and HA-SMAD6 both in the nucleus and cytoplasm (electronic supplementary material, figure S5b). When FLAG-ALK3 was co-expressed with GFP-USP15, a significant GFP-USP15 fluorescence was observed along cytoplasmic membranes, partially overlapping with FLAG-ALK3 expression. FLAG-ALK3 expression was also observed along the cytoplasmic membranes, in the cytoplasm and around the nuclear periphery (electronic supplementary material, figure S5c). When GFP-USP15, FLAG-ALK3 and HA-SMAD6 were all expressed together, a partial reduction in the nuclear GFP-USP15 fluorescence was observed (figure 4*c*; electronic supplementary material, figure S5d,e). The enhanced cytoplasmic membrane fluorescence of GFP-USP15 as well as overall co-localization of GFP-USP15 and FLAG-ALK3 was still observed (figure 4*c*). Partial co-localization between HA-SMAD6 and FLAG-ALK3 as well as GFP-USP15 and HA-SMAD6 was also observed in the cytoplasm (figure 4*c*). These results indicate that SMAD6 does not direct USP15 to ALK3 in the membrane. Rather, expression of SMAD6 possibly modulates USP15 and/or ALK3 in ways that potentially limit ALK3 access to USP15, thereby disrupting the USP15 : ALK3 interaction.

### 3.4. USP15 deubiquitylates ALK3

**Figure 5.**
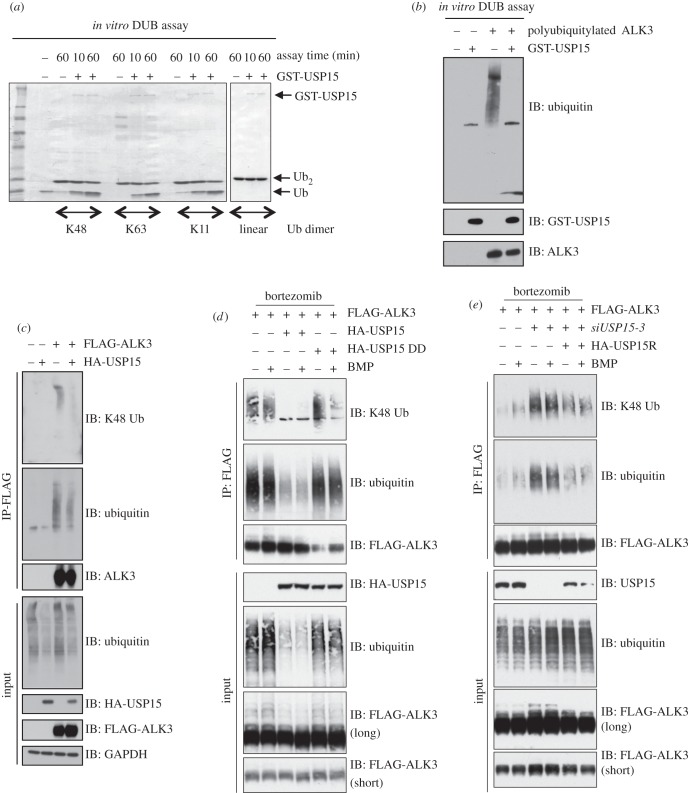
USP15 deubiquitylates ALK3. (*a*) Human recombinant GST-USP15 expressed in *Escherichia coli* was employed in an *in vitro* deubiquitylation assay using K48-, K63- and K11-linked and linear di-ubiquitin (Ub) molecules as substrates. The reactions were quenched by adding SDS sample buffer and boiling for 5 min. The samples were resolved by SDS-PAGE, Coomassie stained and then imaged. (*b*) HEK293 cell transfected with FLAG control or FLAG-ALK3 vectors were treated with bortezomib (10 μM) for 3 h prior to lysis. FLAG-IPs from extracts (1 mg protein) were used as substrates for GST-USP15 in an *in vitro* deubiquitylation assay. The reactions were stopped by adding SDS sample buffer and boiling for 5 min. The samples were resolved by SDS-PAGE and subjected to immunoblotting analysis using the indicated antibodies. (*c*) HEK293 cells were transiently transfected with FLAG control or FLAG-ALK3 vectors with or without HA-USP15. Prior to lysis, cells were treated with 10 μM bortezomib for 3 h. FLAG-IPs and extract inputs were resolved by SDS-PAGE and subjected to immunoblotting analysis using the indicated antibodies. (*d*) HEK293 cells transiently expressing FLAG-ALK3, HA-USP15 and USP15 C269S DUB dead mutant (DD) were serum-starved overnight, pretreated with 10 μM bortezomib for 3 h then stimulated with 6.25 ng ml^–1^ BMP for 1 h prior to lysis. FLAG-IPs and extract inputs were resolved by SDS-PAGE and subjected to immunoblotting with the indicated antibodies. (*e*) HEK293 cells transiently expressing *siUSP15-3*, FLAG-ALK3 and *siUSP15-3* resistant silent mutant of HA-USP15 (HA-USP15R) were serum-starved overnight, pretreated with 10 μM bortezomib for 3 h then stimulated with 6.25 ng ml^−1^ BMP for 1 h prior to lysis. FLAG-IPs and extract inputs were resolved by SDS-PAGE and subjected to immunoblotting with the indicated antibodies.

The strong interaction between USP15 and ALK3 and their co-localization at the plasma membrane suggested that USP15 could act as a DUB for ALK3. Human recombinant USP15 expressed in bacteria displays DUB activity *in vitro* and is capable of cleaving not only K48-linked but also K63- and K11-linked diubiquitin chains (figure 5*a*). It did not cleave linear diubiquitin chain (figure 5*a*). To test whether USP15 can deubiquitylate polyubiquitylated ALK3 *in vitro*, we immunoprecipitated FLAG-ALK3 from HEK293 cells treated with the proteasome inhibitor bortezomib (to enrich the pool of polyubiquitylated FLAG-ALK3) and subjected the FLAG-IPs to *in vitro* deubiquitylation by GST-USP15 (figure 5*b*). In the absence of USP15, FLAG-ALK3 IPs displayed robust polyubiquitylation, while the introduction of GST-USP15 caused efficient deubiquitylation, leading to the accumulation of mono-ubiquitin (figure 5*b*).

We next asked whether USP15 can deubiquitylate ALK3 in cells. HEK293 cells were transfected with either a FLAG-control vector or a vector encoding FLAG-ALK3 in the presence or absence of HA-USP15 (figure 5*c*). In the absence of HA-USP15, efficient K48-linked polyubiquitin and ubiquitin chains were observed in FLAG-ALK3 IPs but not in control FLAG-IPs (figure 5*c*). This polyubiquitylation does not appear to require ALK3 kinase activity, as a catalytically inactive ALK3-D380A mutant was polyubiquitylated to a similar extent as the wild-type ALK3 (electronic supplementary material, figure S6a). Both K48-linked polyubiquitin and total ubiquitin chains were significantly reduced in FLAG-ALK3 IPs from cells transfected with HA-USP15 (figure 5*c*). Interestingly, the level of overall polyubiquitylation in extract inputs was also reduced when wild-type HA-USP15 was overexpressed (figure 5*c*).

To ask whether FLAG-ALK3 deubiquitylation requires the deubiquitylase activity of USP15, we tested the ability of a catalytically inactive mutant of USP15 (USP15[C269S]; USP15-DD) to deubiquitylate FLAG-ALK3 in HEK293 cells. As described earlier, in the absence of HA-USP15 overexpression, FLAG-ALK3 IPs displayed robust polyubiquitylation, particularly the K48-linked polyubiquitylation (figure 5*d*). The polyubiquitylation of FLAG-ALK3 was further confirmed in extracts by the observations of higher mobility bands detected by anti-FLAG antibody immunoblotting (figure 5*d*; FLAG-ALK3 long). Treatment of cells with BMP did not alter the levels of FLAG-ALK3 polyubiquitylation (figure 5*d*). Overexpression of wild-type HA-USP15 but not HA-USP15-DD resulted in almost complete loss of polyubiquitylation in FLAG-ALK3 IPs, indicating that the loss of ubiquitylation in FLAG-ALK3 requires the deubiquitylase activity of HA-USP15 (figure 5*d*).

As noted earlier (figure 5*c*), the overall levels of polyubiquitin chains in extracts were significantly reduced by overexpression of wild-type HA-USP15 but were not affected by HA-USP15-DD (figure 5*d*). This global reduction caused by overexpression of USP15 is consistent with its ability to deubiquitylate multiple ubiquitin linkage types *in vitro* (figure 5*a*). To establish a role for endogenous USP15 in deubiquitylating ALK3, a loss-of function experiment was performed (figure 5*e*). FLAG-ALK3 was transfected into HEK293 cells in which endogenous USP15 was depleted with *siRNA*. Depletion of endogenous USP15 led to an increase in K48-linked polyubiquitylation in FLAG-ALK3 IPs as well as in overall polyubiquitylation (figure 5*e*). This increased polyubiquitylation was significantly inhibited when cells were transfected with *siRNA*-resistant mutant of HA-USP15 (figure 5*e*), suggesting that the observed effects were unlikely to be due to off-target effects of USP15 *siRNA*. Treatment of cells with BMP had little effect on levels of FLAG-ALK3 polyubiquitylation, indicating that USP15 regulates ALK3 levels even under basal conditions (figure 5*e*).

### 3.5. Polyubiquitylated ALK3 undergoes proteasomal degradation

**Figure 6.**
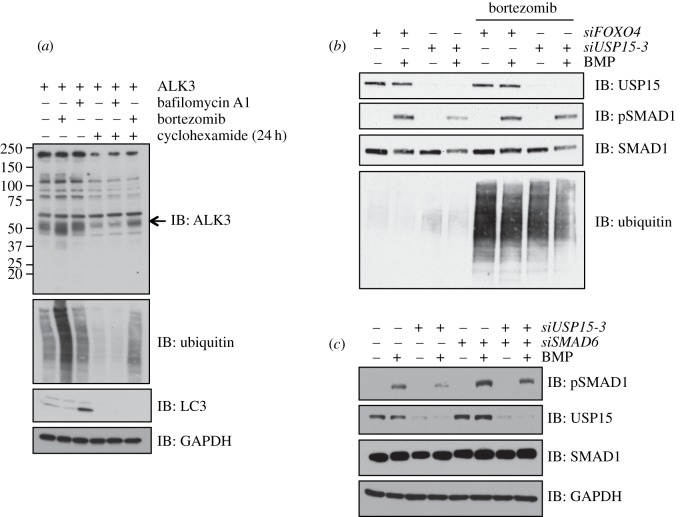
ALK3 undergoes proteasomal degradation. (*a*) HEK293 cells transfected with untagged ALK3 were treated with or without 20 μM cycloheximide for 24 h prior to lysis. Cells were treated with DMSO control, 100 nM bafilomycin A1 (to inhibit vacuolar-type H+ ATPase) or 10 μM bortezomib (to inhibit the proteasome) for 3 h prior to lysis. Extracts were resolved by SDS-PAGE and subjected to immunoblotting with the indicated antibodies. (*b*) HEK293 cells were transiently transfected with *siFoxO4* or *siUSP15-3*. Cells were serum-starved overnight, treated with or without 10 μM bortezomib for 3 h and then stimulated with or without 6.25 ng ml^−1^ BMP for 1 h prior to lysis. Extracts were resolved by SDS-PAGE and subjected to immunoblotting with antibodies against endogenous USP15, pSMAD1 and total SMAD1. (*c*) HEK293 cells were transiently transfected with *siFoxO4* (–), *siUSP15-3* or *siSMAD6* as indicated. Twenty-four hours post *siRNA* transfection, cells were serum-starved overnight and stimulated with or without 6.25 ng ml^−1^ BMP for 1 h prior to lysis. Extracts were resolved by SDS-PAGE and subjected to immunoblotting with antibodies against pSMAD1, total SMAD1, USP15 and GAPDH. The SMAD6 knockdown was confirmed by qRT-PCR (electronic supplementary material, figure S7).

Polyubiquitylation-dependent destruction of ALK3 in cells could be mediated by the proteasomal or lysosomal degradation pathways [30] or even both [31]. To test these possibilities, we investigated the turnover of untagged human ALK3 expressed in HEK293 cells in the presence of the proteasomal inhibitor bortezomib and lysosomal fusion inhibitor bafilomycin [25,32–34]. As expected, Bortezomib resulted in the accumulation of polyubiquitin chains in extracts, whereas bafilomycin yielded increased levels of LC3-II [32] (figure 6*a*). Treatment of cells with bortezomib resulted in enhanced levels of ALK3 compared with control, whereas bafilomycin did not (figure 6*a*). Similarly, when cells were treated with cycloheximide for 24 h prior to lysis to prevent de novo ALK3 synthesis, bortezomib but not bafilomycin resulted in enhanced levels of ALK3 compared with control (figure 6*a*). Analogous results were obtained when *Xenopus* ALK3-HA was expressed in HEK293 cells (electronic supplementary material, figure S6a). Consistently, the pretreatment of cells with bortezomib but not bafilomycin resulted in enhanced levels of polyubiquitylation in FLAG-ALK3 IPs (electronic supplementary material, figure S6b). Together, these results suggest that ALK3 polyubiquitylation leads to its proteasomal degradation.

### 3.6. *siUSP15*-mediated inhibition of bone morphogenetic protein signalling is rescued by bortezomib and SMAD6 depletion

Depletion of USP15 inhibits BMP-induced SMAD1 phosphorylation and promotes K48-linked polyubiquitylation of ALK3 (figures 2*a* and 5*e*). If this depends upon polyubiquitylation-mediated proteosomal degradation of ALK3, then proteasomal inhibition predicted to stabilize ALK3 should rescue the effect of USP15 depletion on BMP signalling. Consistent with this, pretreatment of HEK293 cells with the proteasomal inhibitor bortezomib does indeed rescue BMP-induced pSMAD1 levels reduced by USP15 depletion (figure 6*b*).

As discussed earlier, SMAD6 recruits the E3 ubiquitin ligases that target ALK3 for polyubiquitin-mediated proteasomal degradation [18]. We therefore asked whether *siRNA*-mediated knockdown of SMAD6 was also able to rescue the inhibition of BMP signalling caused by USP15 depletion. In HEK293 cells, the depletion of SMAD6 alone resulted in enhanced phosphorylation of SMAD1 in response to BMP signalling (figure 6*c*). More significantly, loss of SMAD6 expression partially rescued the reduction in BMP-induced pSMAD1 caused by USP15 depletion (figure 6*c*). In the absence of antibodies to detect endogenous expression of SMAD6, the SMAD6 knockdown was confirmed by qRT-PCR (electronic supplementary material, figure S7).

### 3.7. USP15 knockdown inhibits alkaline phosphatase activity

**Figure 7.**
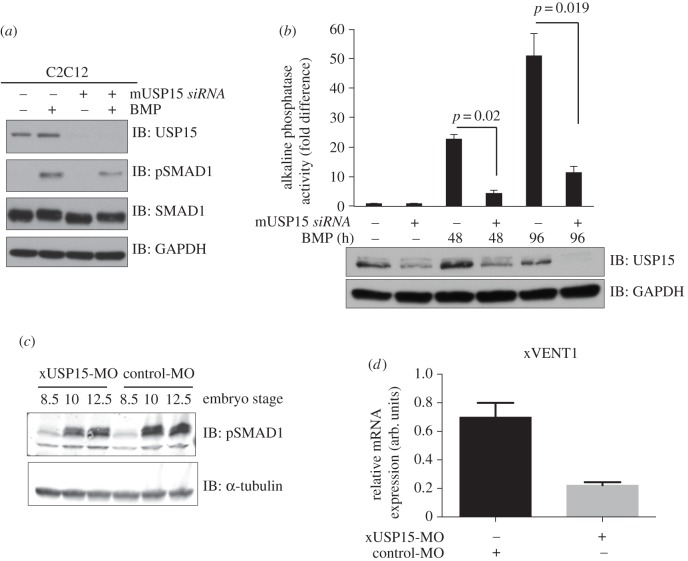
USP15 impacts osteoblastic differentiation in C2C12 myoblasts and modulates BMP signalling in *Xenopus* embryogenesis. (*a*) Mouse myoblast cell line C2C12 were transfected with *siRNAs* targeting mouse FoxO4 or USP15. Cells were serum-starved overnight and treated with or without BMP for 1 h prior to lysis. Extracts were resolved by SDS-PAGE and immunoblotted with antibodies against USP15, pSMAD1, total SMAD1 and GAPDH. (*b*) C2C12 cells transfected with mouse *siFoxO4* or mouse *siUSP15* were grown for up to 4 days in the presence of BMP. Cells were lysed and the alkaline phosphatase activity measured using a fluorescence plate reader. Data are represented as mean of three biological replicates and error bars indicate s.d. Representative extracts were resolved by SDS-PAGE and subjected to immunoblotting with antibodies against USP15 and GAPDH. (*c*) *Xenopus* embryos were injected with 80 ng of either xUSP15- (xUSP15-MO) or control-MO morpholinos at the one-cell stage and then collected at the indicated stages. Lysates were resolved by SDS-PAGE and immunoblotted with antibodies against pSMAD1 and α-tubulin. (*d*) qRT-PCR analysis of *xVENT1* mRNA expression. Embryos were injected with 80 ng of either USP15-MO or control-MO at the one-cell stage and then animal caps were cut at stage 8.5. The animal caps were collected at the equivalent embryo stage of 10.5 and processed for qRT-PCR.

BMP signalling plays a key role in inducing myoblast progenitor differentiation to the osteoblast lineage, as marked by the acquisition of alkaline phosphatase activity [35]. We investigated the effects of USP15 depletion on BMP-induced SMAD1 phosphorylation and the development of alkaline phosphatase activity in mouse myoblast C2C12 cells [26,35]. *RNAi*-mediated depletion of USP15 in C2C12 cells resulted in a more than 90% reduction in USP15 protein expression (figure 7*a*). Under these conditions, BMP-induced phosphorylation of SMAD1 was significantly reduced compared with controls (figure 7*a*), a reduction that was not due to a decrease in total levels of SMAD1 (figure 7*a*). Loss of USP15 significantly reduced BMP-induced alkaline phosphatase activity in C2C12 cells at both 48 h and 96 h post-BMP stimulation (figure 7*b*).

### 3.8. USP15 impacts bone morphogenetic protein signalling during *Xenopus* embryogenesis

BMP signalling plays a crucial role in dorsal–ventral patterning during *Xenopus* development [3]. BMP-mediated phosphorylation of SMAD1 in *Xenopus* embryos is detected after stage 9 of development and is sustained thereafter [26]. We investigated the role of USP15 on BMP signalling during *Xenopus* embryogenesis. Injection of antisense morpholino oligonucleotides targeting *Xenopus* USP15 (xUSP15-MO) into one-cell-stage embryos caused no discernible change in the level of pSMAD1 at stage 8.5 but caused a significant reduction in pSMAD1 levels at subsequent developmental stages (10 and 12.5) compared with embryos injected with control morpholinos (control-MO; figure 7*c*). However, the injection of xUSP15-MO did not affect the stability of SMAD1 at any developmental stage when compared with control-MO (electronic supplementary material, figure S8a–c), suggesting that USP15 does not act on SMAD1 directly but probably acts upstream of SMAD1. Consistent with this, injection of one-cell-stage embryos with xUSP15-MO resulted in the reduction of *Xenopus* ALK3 levels over control (electronic supplementary material, figure S8d). By contrast, overexpression of human USP15 resulted in the stabilization of xALK3 (electronic supplementary material, figure S8d). We also investigated the effect of USP15 on the expression of the BMP marker *xVENT1* in embryos. Animal caps from embryos injected with either xUSP15-MO or control-MO were cut at stage 8.5, and then harvested for RNA at stage 10. Injection of USP15-MO alone resulted in a significant depletion of BMP-induced *xVENT1* mRNA expression over control (figure 7*d*).

## 4. Discussion

We show that USP15 interacts with SMAD6 and ALK3 and enhances BMP signalling by deubiquitylating ALK3 and rescuing it from proteasomal destruction. Depletion of USP15 reduces BMP-induced phosphorylation of SMAD1 and the transcription of BMP-target genes, as well as inhibiting the differentiation of myoblasts into osteoblasts. Furthermore, we demonstrate that ablation of USP15 expression from *Xenopus* embryos results in the inhibition of BMP signalling *in vivo*.

### 4.1. Reversible ubiquitylation of type I receptors is key to fine-tuning of bone morphogenetic protein signalling

The regulation of ALK3 polyubiquitylation mediated by E3 ubiquitin ligases recruited by SMAD6 has been reported previously [14,18]. Polyubiquitylation of certain receptor kinases, such as EGFR, causes endocytosis-mediated degradation via the lysosome, which can be inhibited by bafilomycin A1, a potent inhibitor of lysosomal acidification [33,36]. Our findings demonstrate that bafilomycin A1 has no effect on ALK3 turnover, while the proteasome inhibitor bortezomib suppresses ALK3 degradation. These results suggest that ALK3 turnover in cells is controlled primarily by proteasomal degradation. This is consistent with our observations of K48-linked ubiquitin chains, known to promote proteasomal degradation, on ALK3 IPs. The polyubiquitylation of ALK3 overexpressed in HEK293 cells was unaffected by BMP stimulation for 1 h. Because the accumulation of SMAD6 and associated E3 ubiquitin ligases upon BMP treatment takes substantially longer, it would be interesting to see whether ALK3 polyubiquitylation is enhanced by a longer course of BMP treatment. A catalytically inactive ALK3 mutant overexpressed in HEK293 cells was polyubiquitylated to the same extent as the wild-type, suggesting that the kinase activity is not essential for ALK3 ubiquitylation.

Recent studies have highlighted the importance of deubiquitylation of type I receptors in the dynamic fine-tuning of the TGFβ signalling pathway [14,17,20–23,25]. For example, the closely related DUBs USP4, USP11 and USP15 have all been implicated in the control of TGFβ signalling through deubiquitylation of the type I TGFβ receptor ALK5 [20–22]. The DUBs that act on type I receptors in the BMP pathway, however, have not been characterized. Here, we demonstrate that USP15 enhances BMP signalling by associating with and deubiquitylating the BMP type I receptor ALK3. Moreover, we show that USP15 interacts with other type I BMP receptors ALK2 and ALK6, as well as TGFβ receptor ALK5. Co-expression of FLAG-ALK3 and GFP-USP15 causes their co-localization, especially in the plasma membrane. USP15 has also been reported to bind to and deubiquitylate monoubiquitylated R-SMADs to activate TGFβ/BMP signalling [23]. However, we were not able to detect an interaction between USP15 and R-SMADs and, consistent with this, depletion or overexpression of USP15 from human and mouse cells as well as *Xenopus* embryos did not cause significant changes in the levels of endogenous SMAD1.

Among the DUBs, USP4, USP11 and USP15 are very similar, and they probably have similar cellular targets. Pathway specificity is likely to be conferred by the relative affinities with which they bind particular type I TGFβ/BMP receptors and/or I-SMADs, although it is likely that other factors are important. We demonstrate that USP15 interacts with type I BMP receptor ALKs 2, 3 and 6 as strongly as it binds to type I TGFβ receptor ALK5. SMAD6 overexpression disrupts the association of USP15 with ALK3 and potently inhibits BMP signalling. On the other hand, while SMAD6 also interacts with USP11 strongly, depletion of USP11 did not inhibit BMP signalling. Similarly, USP4 does not affect BMP signalling [22]. The activity and substrate specificity of DUBs might also be influenced by post-translational modifications within DUBs or their targets [25], and differential expression of these DUBs in cells and tissues could also contribute to the context-dependent fine-tuning of TGFβ/BMP signalling. Targeted disruption of individual DUBs in mice might shed molecular insights into the pathway-selective nature of USP4, USP11 and USP15.

### 4.2. USP15 impacts bone morphogenetic protein signalling in multiple species

USP15 loss-of-function studies confirm that this DUB plays a role in BMP signalling in several human and mouse cell lines as well as *Xenopus* embryos. In human HEK293, HeLa and U2OS cells, and in mouse C2C12 cells, *RNAi*-mediated depletion of USP15 resulted in inhibition of BMP-induced phosphorylation of SMAD1. Depletion of USP15 from HEK293 cells caused a reduction in BMP-induced transcription, whereas depletion of USP11 did not. Similarly, BMP-induced transcription was unaffected following depletion of USP4 [22]. Reduction of USP15 expression from C2C12 myoblast cells inhibited BMP-induced osteoblastic differentiation, as judged by the reduction in alkaline phosphatase induction. Similarly, loss of USP15 expression in *Xenopus* embryos led to the reduction in pSMAD1 levels as well as the expression of the ventral marker *xVENT1.* A genome wide loss-of-function screen on all zebrafish deubiquitylases also identified USP15 as a critical player in dorsal–ventral patterning through the BMP pathway [37].

### 4.3. USP15 as a potential target for the development of inhibitors of bone morphogenetic protein and transforming growth factor beta signalling

The beneficial roles for putative USP15 inhibitors against TGFβ signalling associated pathologies, such as glioblastoma, have been discussed previously [21]. Here, our findings show that USP15 DUB activity is essential for BMP signalling, and that loss of USP15 function inhibits BMP signalling in human and mouse cells, as well as during *Xenopus* embryogenesis. Mutations leading to overactive BMP signalling are associated with diseases such as heterotopic ossification, Duchenne muscular dystrophy and bone metastasis [6,38]. Moreover, high alkaline phosphatase activity is linked to poor prognosis in patients with prostatic bone metastases [38], and we found that loss of USP15 inhibited BMP-induced alkaline phosphatase activity in C2C12 cells. Inhibition of USP15 might therefore be employed as a strategy to inhibit BMP signalling. However, as would be expected for any DUB, USP15 has multiple other reported targets [39–43] and is likely to have many more; hence USP15 inhibitors will be predicted to be non-selective and affect many cellular processes beyond those controlled by BMP/TGFβ signals.

## 5. Material and methods

### 5.1. Antibodies

Antibodies against USP11, USP15 and SMAD1 were raised in sheep using GST-tagged proteins as antigens and affinity purified. Anti-SMAD1 antibody for use with *Xenopus* extracts was from Santa Cruz Biotechnology. Antibody against ALK3 was raised in sheep using His-ALK3(aa200-end) as an antigen and affinity purified. Anti-HA-HRP antibody and anti-α tubulin were from Sigma. Antibodies against phospho-SMAD1/5 (Ser463/465) and 8 (Ser426/428), GAPDH, β-actin and Lamin A/C were from Cell Signaling Technology. Anti-ubiquitin antibody was from Dako. Goat anti-rabbit, mouse and sheep HRP conjugated antibodies were from Pierce. Goat anti-mouse and anti-rabbit IRDye 680LT and 800CW coupled antibodies were from Li-Cor.

### 5.2. Plasmids

Mammalian expression constructs encoding human USP11, USP15, ALK2, ALK3, ALK3[D380A], ALK5, ALK6, SMAD1, 2, 3, 4, 6 and 7 were cloned into pCMV5 or pCDNA-Frt-TO (Invitrogen) vectors with or without N-terminal 3xFLAG, HA and GFP-tags. pCDNA-Frt-TO plasmids were used to generate stable tetracycline-inducible HEK293 cell lines following the manufacturer's protocol (Invitrogen). All DNA constructs used were verified by DNA sequencing, performed by DNA Sequencing & Services (MRCPPU, College of Life Sciences, University of Dundee, UK, www.dnaseq.co.uk) using Applied Biosystems Big-Dye v. 3.1 chemistry on an Applied Biosystems model 3730 automated capillary DNA sequencer. *Xenopus* ALK3 (xALK3-HA) was constructed by inserting the 3xHA tag 3′ to the ALK3 signal sequence by PCR using the pSP64TBMPR plasmid as a template (Addgene #15068) [44] with the following primers: (Forward: GTACCTGACTATGCATACCCTTATGATGTACCAGACTACGCTCAGGACTTTAACATCTTGCCACACAGAAC; reverse: GTCATAAGGATAAGCGTAATCTGGAACATCGTATGGGTATCCTTGGGTATGAATAACAAGCAGTAAG). The resulting PCR product was phosphorylated with polynucleotide kinase and then ligated with DNA ligase followed by DpnI digest.

### 5.3. Cell culture, transfection and lysis

Cells were propagated in DMEM media (Gibco) supplemented with 10% FBS (Hyclone), 1% penicillin/streptomycin (Gibco) and 2 mM l-glutamine. Cells were kept at 37°C in a humidified incubator with 5% CO_2_. Cell lines stably expressing tetracycline-inducible GFP-tagged proteins were grown in media that additionally contained 100 µg ml^−1^ hygromycin and 15 µg ml^−1^ blasticidin. Human embryonic kidney (HEK293) cells were transfected with appropriate constructs (2 µg of each plasmid per 10-cm diameter dish) using polyethyleneimine (PEI; Polysciences) as described previously [45]. For *siRNA* transfections, Transfectin reagent (Bio-Rad) was used following the manufacturer's protocol. Unless indicated otherwise, cells were treated with 6.25 ng ml^−1^ BMP-2 for 1 h, 100 nM bafilomycin A1 for 3 h, 10 µM bortezomib for 3 h or control solvents prior to lysis. For protein applications, cells were scraped directly into cell lysis buffer (50 mM Tris–HCl pH 7.5, 1 mM EGTA, 1 mM EDTA, 1% Triton X-100, 1 mM activated sodium orthovanadate, 50 mM sodium flouride, 5 mM sodium pyrophosphate, 0.27 M sucrose, 5 mM β-glycerophosphate, 0.1% β-mercaptoethanol and 1 tablet of protease inhibitor cocktail (Roche) per 25 ml) and snap frozen in liquid nitrogen. For RNA applications, cells were processed using an RNA extraction kit (Qiagen) according to the manufacturer's instructions.

### 5.4. *Xenopus* maintenance and manipulation

*Xenopus* embryos were obtained by *in vitro* fertilization and staged according to Nieuwkoop & Faber [46]. Lissamine coupled USP15 (xUSP15-MO) and control antisense morpholino (control-MO) oligonucleotides [23] were obtained from GeneTools (Philomath, OR, USA). These were dissolved in distilled water and stored at 4°C. Sequences were as follows: xUSP15-MO: 5′-CGCCCTCCGCCATCTTACTCACTT-3′ Lissamine; control-MO 5′-CCTCTTACCTCAGTTACAATTTATA-3′ Lissamine. Animal cap assays were carried out as described previously [47].

### 5.5. Immunoprecipitation and immunoblotting

Snap frozen cell extracts were allowed to thaw on ice and centrifuged at 17 500*g* for 10 min at 4°C. Protein concentration was determined spectrophotometrically using Bradford reagent (Thermo Scientific). Extracts (1 mg) were then subjected to immunoprecipitation with 10 μl packed beads (GFP-Trap (Chromatek), anti-FLAG M2 gel (Sigma) or 2 μg specific antibody or pre-immune IgG bound to Protein G Sepharose beads (GE Healthcare)) by rotating for 2 h at 4°C. Protein-bound beads were then washed twice in lysis buffer with 0.5 M NaCl, and twice in buffer A (50 mM Tris–HCl pH 7.5, 0.1 mM EGTA, 0.1% β-mercaptoethanol) at 4°C. Samples were then reduced in 50 μl of 1× SDS sample buffer (50 mM Tris–HCl pH 6.8, 2% SDS, 10% glycerol, 0.02% bromophenol blue, 1% β-mercaptoethanol) and boiled at 95°C for 5 min. Cell extract inputs (20 μg protein unless stated otherwise) or IPs (40% unless stated otherwise) were resolved on 10% denaturing SDS polyacrylamide gels and transferred onto a nitrocellulose membrane (Whatman). Immunoblot analysis on membranes was performed as described previously [20]. For *Xenopus* experiments, embryos were cultured in the presence or absence of 10 µM cycloheximide (Sigma) in 0.1× NAM (Normal Amphibian Medium [48]) for indicated times. Ten embryos per time point were lysed in 100 µl of PhosphoSafe reagent (Novogen) supplemented with complete protease inhibitor (Roche), and then extracted with an equal volume of FREON (Sigma) to remove yolk proteins. Samples were reduced by adding 4× SDS sample buffer (Li-Cor) with 10% β-mercaptoethanol and then boiled for 5 min. For immunoprecipitations of xAlk3-HA, 30 embryos/condition were lysed in PBSCA (PBS, 1% Igepal CA-630 (Sigma), 10 µg ml^–1^ leupeptin (Roche), 10 µg ml^−1^ aprotinin (Roche), 1 mM *N*-ethylmaleimide (Sigma), 100 nM 1,10-phenanthroline (Sigma), complete protease inhibitor (Roche)), and then cleared by centrifugation for 20 min at 14 000*g* at 4°C. Mouse anti-HA (HA-7, Sigma) was added to lysates and then incubated on a rotator for 2 h at 4°C followed by an overnight incubation on a rotator with 15 µl of Dynabeads Protein G (Invitrogen) at 4°C. Protein-bound beads were washed 5× with PBSCA. Samples were then reduced in 40 µl of 1× SDS sample buffer with 2.5% β-mercaptoethanol and boiled for 5 min. Samples were separated on either 4–20% acrylamide gels (NuSep) or 7.5% TGX gels (Bio-Rad) and then transferred to Immobilon-FL PVDF membranes (Millipore). Membranes were blocked for 1 h at room temperature (RT) in Li-Cor Blocking Buffer, and then incubated in primary antibody in PBST (PBS, 0.1% Tween 20) overnight at 4°C. Blots were washed 3× in PBST then incubated with a combination of IRDye 680LT and 800CW labelled secondary antibodies (1 : 15 000 in PBST supplemented with 0.02% SDS) for 1 h at RT. Washed blots were imaged with a Li-Cor Odyssey scanner followed by image analysis using Image Studio (Li-Cor).

### 5.6. Mass-spectrometric analysis

Mass-spectrometric analysis on GFP-IPs was performed by LC–MS–MS using a linear ion trap–orbitrap hybrid mass spectrometer (LTQ-Orbitrap, Thermo Fisher Scientific) equipped with a nanoelectrospray ion source (Thermo) and coupled to a Proxeon EASY-nLC system as described previously [49].

### 5.7. *In vitro* ubiquitylation and deubiquitylation assays

For cleavage of linear, K11-, K48- and K63-linked di-ubiquitin, 36 nM GST-USP15 was added to a 3 nM of each ubiquitin dimer. The cleavage reactions (20 μl) were carried out at RT in a solution containing 50 mM Tris–HCl pH 7.5, 100 mM NaCl and 5 mM DTT. Reactions were stopped at 10 or 60 min by the addition of SDS sample buffer. The cleavage of ubiquitin dimers was visualized by resolving samples with SDS-PAGE and Coomassie staining the gel. The *in vivo* and *in vitro* deubiquitylation assays of polyubiquitylated ALK3 were performed as previously described [25]. In brief, in-cell deubiquitylation assays were performed in HEK293 cells by co-transfecting FLAG-ALK3, and HA-USP15 constructs. Prior to lysis cells were treated with 10 µM bortezomib for 3 h and FLAG was immunoprecipitated. The *in vitro* DUB assay of *in vivo* polyubiquitylated FLAG-ALK3 IPs was performed with GST-USP15 in DUB assay buffer for 1 h at 30°C on an IP-shaker. Proteins were resolved by SDS-PAGE and immunoblotted with the indicated antibodies.

### 5.8. *RNAi* and quantitative PCR

The siRNA and qRT-PCR primer sequences used in this study are as follows:

All *siRNAs* were purchased from Sigma. Human *siRNAs* targeting USP11: *siUSP11* (5′–3′): GAUUCUAUUGGCCUAGUAU. Human siRNAs against USP15: *siUSP15-1* (5′–3′): CUCUUGAGAAUGUGCCGAU; *siUSP15-2*: CACAAUAGAUACAAUUGAA; and *siUSP15-3* CACAUUGAUGGAAGGUCAA. Control FoxO4 *siRNAs* (5′–3′): human: CCCGACCAGAGAUCGCUAA, mouse: GCAAGUUCAUCAAGGUUCA. Mouse siRNAs targeting USP15: *siUSP15#1* (5′–3′): GAACUACUGGCUUUCCUGU; #2: CCUUAUUGAUGAGUUGGAU #3: GGUAUUGUCCAAAUUGUAA.

Human qRT-PCR primers used were as follows

GAPDH: (F,R) (ATCTTCTTTTGCGTCGCCAG, GCTGAGACACCATGGGGAA)
FoxO4: (F,R) (TTGGAGAACCTGGAGTATGTGACA, AAGCTTCCAGGCATGACTCAG)
USP11: (F,R) (GTGTTCAAGAACAAGGTTGG, CGATTAAGGTCCTCATGCAG)
USP15: (F,R) (GACCCATTGATAACTCTGGAC, TGTTCAACCACCTTTCGTG)
ID1: (F,R) (AGGCTGGATGCAGTTAAGGG, GACGATCGCATCTTGTGTCG)
SMAD6: (F, R) (CCATCAAGGTGTTCGACTTC, TTGTTGAGGAGGATCTCCAG)
PAI1: (F, R) (AGCTCCTTGTACAGATGCCG, ACAACAGGAGGAGAAACCCA)

The qRT-PCR reactions were performed in triplicate on an iQ5 PCR machine (Bio-Rad) and data analysed using Microsoft Excel. All experiments have a minimum *n* = 3. Error bars represent the standard deviation. Statistical comparisons (*p*-values) were obtained from Student's *t*-test as described previously [25].

*Xenopus* qRT-PCR primers used were as follows:

H4 (F,R) (CGGGATAACATTCAGGGTATCACT, ATCCATGGCGGTAATGTCTTCCT); xVent1 (F,R) (TTCCCTTCAGCATGGTTCAAC, GCATCTCCTTGGCATATTTGG). qRT-PCR reactions were carried out on a Roche Lightcycler 480 using LightCycler 480 SYBR Green I master mix (10 µl reactions) according to the manufacturer's instructions.

### 5.9. Alkaline phosphatase assay

C2C12 cells were transfected with *siRNAs* against mouse USP15 or mouse FoxO4 (300 pM each) using transfectin reagent and grown in DMEM with 5% FBS. Forty-eight hours post-transfection 100 ng ml^−1^ BMP2 was added for 2–4 days, and cells were lysed using CelLytic reagent (Sigma). The protein concentration was determined using Bradford and equal amounts were used to detect alkaline phosphatase activity. Alkaline phosphatase detection was carried out in accordance with the manufacturer's protocol (Sigma). In brief, cell extracts were diluted in assay buffer and fluorescent substrate (4-methylumbelliferyl phosphate disodium salt) was added. Fluorescence was detected using a fluorescent plate reader (PHERAstar) at 350 nm excitation and 460 nm emission.

### 5.10. Immunofluorescence

Human osteosarcoma U2OS cells transfected with FLAG-ALK3, HA-SMAD6 and/or GFP-USP15 were seeded onto poly-l-lysine treated glass coverslips in 6-well culture dishes. Cells were washed in PBS before fixation with 3.7% paraformaldehyde for 20 min at RT. The coverslips were washed a further three times before permeabilization with 0.2% Triton X-100 in PBS for 15 min at RT. Cells were rinsed with PBS before being incubated for 1 h in blocking solution (5% (v/v) normal donkey serum, 0.01% (v/v) fish skin gelatin, 0.1% (v/v) Triton X-100, 0.05% (v/v) Tween-20 in PBS). Primary antibody (HA, Roche; FLAG, Sigma; GFP, Life Technologies) incubation was done for 16 h in a humidified chamber at 4°C. After thorough washes in PBS, cells were incubated with AlexaFluor and Cyanine Cy5 (Jackson ImmunoResearch Labs) secondary antibodies for 1 h in the dark. Cells were washed three more times in PBS and once with deionized water before being mounted onto glass slides using ProLong Gold mounting reagent (Life Technologies), which contained the nuclear stain 4′,6-diamidino-2-phenylindole. Slides were viewed using a Nikon Eclipse Ti microscope fitted with a 60× lens and a cooled charge-coupled device camera.

## Supplementary Material

Supplementary Figure Legends and Data

## References

[1] Cai J Pardali E Sanchez-Duffhues G ten Dijke P 2012 BMP signaling in vascular diseases. FEBS Lett. 586, 1993–2002. (doi:10.1016/j.febslet.2012.04.030) 22710160 10.1016/j.febslet.2012.04.030

[2] Chen D Zhao M Mundy GR 2004 Bone morphogenetic proteins. Growth Factors 22, 233–241. (doi:10.1080/08977190412331279890) 15621726 10.1080/08977190412331279890

[3] De Robertis EM Kuroda H 2004 Dorsal-ventral patterning and neural induction in *Xenopus* embryos. Annu. Rev. Cell Dev. Biol. 20, 285–308. (doi:10.1146/annurev.cellbio.20.011403.154124) 15473842 10.1146/annurev.cellbio.20.011403.154124 PMC2280069

[4] Harland R 2000 Neural induction. Curr. Opin. Genet. Dev. 10, 357–362. (doi:10.1016/S0959-437X(00)00096-4) 10889069 10.1016/s0959-437x(00)00096-4

[5] Harradine KA Akhurst RJ 2006 Mutations of TGFβ signaling molecules in human disease. Ann. Med. 38, 403–414. (doi:10.1080/07853890600919911) 17008304 10.1080/07853890600919911

[6] Shi S de Gorter DJ Hoogaars WM t Hoen PA ten Dijke P 2013 Overactive bone morphogenetic protein signaling in heterotopic ossification and Duchenne muscular dystrophy. Cell Mol. Life Sci. 70, 407–423. (doi:10.1007/s00018-012-1054-x) 22752156 10.1007/s00018-012-1054-x PMC3541930

[7] Varga AC Wrana JL 2005 The disparate role of BMP in stem cell biology. Oncogene 24, 5713–5721. (doi:10.1038/sj.onc.1208919) 16123804 10.1038/sj.onc.1208919

[8] Bruce DL Sapkota GP 2012 Phosphatases in SMAD regulation. FEBS Lett. 586, 1897–1905. (doi:10.1016/j.febslet.2012.02.001) 22576046 10.1016/j.febslet.2012.02.001

[9] Shi Y Massague J 2003 Mechanisms of TGF-beta signaling from cell membrane to the nucleus. Cell 113, 685–700. (doi:10.1016/S0092-8674(03)00432-X) 12809600 10.1016/s0092-8674(03)00432-x

[10] Ikushima H Miyazono K 2012 TGF-β signal transduction spreading to a wider field: a broad variety of mechanisms for context-dependent effects of TGF-β. Cell Tissue Res. 347, 37–49. (doi:10.1007/s00441-011-1179-5) 21618142 10.1007/s00441-011-1179-5

[11] Massague J 2012 TGFβ signalling in context. Nat. Rev. Mol. Cell Biol. 13, 616–630. (doi:10.1038/nrm3434) 22992590 10.1038/nrm3434 PMC4027049

[12] Caja L Kahata K Moustakas A 2012 Context-dependent action of transforming growth factor β family members on normal and cancer stem cells. Curr. Pharm. Des. 18, 4072–4086. (doi:10.2174/138161212802430459) 22630079 10.2174/138161212802430459

[13] Schier AF Talbot WS 2005 Molecular genetics of axis formation in zebrafish. Annu. Rev. Genet. 39, 561–613. (doi:10.1146/annurev.genet.37.110801.143752) 16285872 10.1146/annurev.genet.37.110801.143752

[14] Al-Salihi MA Herhaus L Sapkota GP 2012 Regulation of the transforming growth factor β pathway by reversible ubiquitylation. Open Biol. 2, 120082 (doi:10.1098/rsob.120082) 22724073 10.1098/rsob.120082 PMC3376735

[15] Chen YL Liu B Zhou ZN Hu RY Fei C Xie ZH Ding X 2009 Smad6 inhibits the transcriptional activity of Tbx6 by mediating its degradation. J. Biol. Chem. 284, 23 481–23 490. (doi:10.1074/jbc.M109.007864) 10.1074/jbc.M109.007864 PMC2749122 19561075

[16] Massague J Gomis RR 2006 The logic of TGFβ signaling. FEBS Lett. 580, 2811–2820. (doi:10.1016/j.febslet.2006.04.033) 16678165 10.1016/j.febslet.2006.04.033

[17] Yan X Liu Z Chen Y 2009 Regulation of TGF-β signaling by Smad7. Acta Biochim. Biophys. Sin (Shanghai) 41, 263–272. (doi:10.1093/abbs/gmp018) 19352540 10.1093/abbs/gmp018 PMC7110000

[18] Goto K Kamiya Y Imamura T Miyazono K Miyazawa K 2007 Selective inhibitory effects of Smad6 on bone morphogenetic protein type I receptors. J. Biol. Chem. 282, 20 603–20 611. (doi:10.1074/jbc.M702100200) 10.1074/jbc.M702100200 17493940

[19] Murakami G Watabe T Takaoka K Miyazono K Imamura T 2003 Cooperative inhibition of bone morphogenetic protein signaling by Smurf1 and inhibitory Smads. Mol. Biol. Cell 14, 2809–2817. (doi:10.1091/mbc.E02-07-0441) 12857866 10.1091/mbc.E02-07-0441 PMC165678

[20] Al-Salihi MA Herhaus L Macartney T Sapkota GP 2012 USP11 augments TGFβ signalling by deubiquitylating ALK5. Open Biol. 2, 120063 (doi:10.1098/rsob.120063) 22773947 10.1098/rsob.120063 PMC3390794

[21] Eichhorn PJ 2012 USP15 stabilizes TGF-β receptor I and promotes oncogenesis through the activation of TGF-β signaling in glioblastoma. Nat. Med. 18, 429–435. (doi:10.1038/nm.2619) 22344298 10.1038/nm.2619

[22] Zhang L 2012 USP4 is regulated by AKT phosphorylation and directly deubiquitylates TGF-β type I receptor. Nat. Cell Biol. 14, 717–726. (doi:10.1038/ncb2522) 22706160 10.1038/ncb2522

[23] Inui M 2011 USP15 is a deubiquitylating enzyme for receptor-activated SMADs. Nat. Cell Biol. 13, 1368–1375. (doi:10.1038/ncb2346) 21947082 10.1038/ncb2346

[24] Zhang X Zhang J Bauer A Zhang L Selinger DW Lu CX Ten Dijke P 2013 Fine-tuning BMP7 signalling in adipogenesis by UBE2O/E2–230K-mediated monoubiquitination of SMAD6. Embo J. 32, 996–1007. (doi:10.1038/emboj.2013.38) 23455153 10.1038/emboj.2013.38 PMC3616286

[25] Herhaus L Al-Salihi M Macartney T Weidlich S Sapkota GP 2013 OTUB1 enhances TGFβ signalling by inhibiting the ubiquitylation and degradation of active SMAD2/3. Nat. Commun. 4, 2519 (doi:10.1038/ncomms3519) 24071738 10.1038/ncomms3519 PMC3791481

[26] Sapkota G Alarcon C Spagnoli FM Brivanlou AH Massague J 2007 Balancing BMP signaling through integrated inputs into the Smad1 linker. Mol. Cell 25, 441–454. (doi:10.1016/j.molcel.2007.01.006) 17289590 10.1016/j.molcel.2007.01.006

[27] Heldin CH Miyazono K ten Dijke P 1997 TGF-β signalling from cell membrane to nucleus through SMAD proteins. Nature 390, 465–471. (doi:10.1038/37284) 9393997 10.1038/37284

[28] Hoodless PA Haerry T Abdollah S Stapleton M O'Connor MB Attisano L Wrana JL 1996 MADR1, a MAD-related protein that functions in BMP2 signaling pathways. Cell 85, 489–500. (doi:10.1016/S0092-8674(00)81250-7) 8653785 10.1016/s0092-8674(00)81250-7

[29] Kretzschmar M Liu F Hata A Doody J Massague J 1997 The TGF-β family mediator Smad1 is phosphorylated directly and activated functionally by the BMP receptor kinase. Genes Dev. 11, 984–995. (doi:10.1101/gad.11.8.984) 9136927 10.1101/gad.11.8.984

[30] Bonifacino JS Weissman AM 1998 Ubiquitin and the control of protein fate in the secretory and endocytic pathways. Annu. Rev. Cell Dev. Biol. 14, 19–57. (doi:10.1146/annurev.cellbio.14.1.19) 9891777 10.1146/annurev.cellbio.14.1.19 PMC4781171

[31] Zhao B Wang Q Du J Luo S Xia J Chen YG 2012 PICK1 promotes caveolin-dependent degradation of TGF-β type I receptor. Cell Res. 22, 1467–1478. (doi:10.1038/cr.2012.92) 22710801 10.1038/cr.2012.92 PMC3463259

[32] Allen GF Toth R James J Ganley IG 2013 Loss of iron triggers PINK1/Parkin-independent mitophagy. EMBO Rep. 14, 1127–1135. (doi:10.1038/embor.2013.168) 24176932 10.1038/embor.2013.168 PMC3981094

[33] Yoshimori T Yamamoto A Moriyama Y Futai M Tashiro Y 1991 Bafilomycin A1, a specific inhibitor of vacuolar-type H^+^-ATPase, inhibits acidification and protein degradation in lysosomes of cultured cells. J. Biol. Chem. 266, 17 707–17 712. 1832676

[34] Yamamoto A Tagawa Y Yoshimori T Moriyama Y Masaki R Tashiro Y 1998 Bafilomycin A1 prevents maturation of autophagic vacuoles by inhibiting fusion between autophagosomes and lysosomes in rat hepatoma cell line, H-4-II-E cells. Cell Struct. Funct. 23, 33–42. (doi:10.1247/csf.23.33) 9639028 10.1247/csf.23.33

[35] Katagiri T 1994 Bone morphogenetic protein-2 converts the differentiation pathway of C2C12 myoblasts into the osteoblast lineage. J. Cell Biol. 127, 1755–1766. (doi:10.1083/jcb.127.6.1755) 7798324 10.1083/jcb.127.6.1755 PMC2120318

[36] Ganley IG Wong PM Gammoh N Jiang X 2011 Distinct autophagosomal-lysosomal fusion mechanism revealed by thapsigargin-induced autophagy arrest. Mol. Cell 42, 731–743. (doi:10.1016/j.molcel.2011.04.024) 21700220 10.1016/j.molcel.2011.04.024 PMC3124681

[37] Tse WK Jiang YJ Wong CK 2013 Zebrafish transforming growth factor-β-stimulated clone 22 domain 3 (TSC22D3) plays critical roles in Bmp-dependent dorsoventral patterning via two deubiquitylating enzymes Usp15 and Otud4. Biochim. Biophys. Acta 1830, 4584–4593. (doi:10.1016/j.bbagen.2013.05.006) 23665588 10.1016/j.bbagen.2013.05.006

[38] Jung K Lein M Stephan C Von Hosslin K Semjonow A Sinha P Loening SA Schnorr D 2004 Comparison of 10 serum bone turnover markers in prostate carcinoma patients with bone metastatic spread: diagnostic and prognostic implications. Int. J. Cancer 111, 783–791. (doi:10.1002/ijc.20314) 15252851 10.1002/ijc.20314

[39] Harper S Besong TM Emsley J Scott DJ Dreveny I 2011 Structure of the USP15 N-terminal domains: a β-hairpin mediates close association between the DUSP and UBL domains. Biochemistry 50, 7995–8004. (doi:10.1021/bi200726e) 21848306 10.1021/bi200726e

[40] Isumi Y Hirata T Saitoh H Miyakawa T Murakami K Kudoh G Doi H Ishibashi K Nakajima H 2011 Transgenic overexpression of USP15 in the heart induces cardiac remodeling in mice. Biochem. Biophys. Res. Commun. 405, 216–221. (doi:10.1016/j.bbrc.2011.01.012) 21219870 10.1016/j.bbrc.2011.01.012

[41] Villeneuve NF Tian W Wu T Sun Z Lau A Chapman E Fang D Zhang DD 2013 USP15 negatively regulates Nrf2 through deubiquitination of Keap1. Mol. Cell 51, 68–79. (doi:10.1016/j.molcel.2013.04.022) 23727018 10.1016/j.molcel.2013.04.022 PMC3732832

[42] Vos RM Altreuter J White EA Howley PM 2009 The ubiquitin-specific peptidase USP15 regulates human papillomavirus type 16 E6 protein stability. J. Virol. 83, 8885–8892. (doi:10.1128/JVI.00605-09) 19553310 10.1128/JVI.00605-09 PMC2738190

[43] Xu M Takanashi M Oikawa K Tanaka M Nishi H Isaka K Kudo M Kuroda M 2009 USP15 plays an essential role for caspase-3 activation during Paclitaxel-induced apoptosis. Biochem. Biophys. Res. Commun. 388, 366–371. (doi:10.1016/j.bbrc.2009.08.015) 19665996 10.1016/j.bbrc.2009.08.015

[44] Graff JM Thies RS Song JJ Celeste AJ Melton DA 1994 Studies with a Xenopus BMP receptor suggest that ventral mesoderm-inducing signals override dorsal signals *in vivo*. Cell 79, 169–179. (doi:10.1016/0092-8674(94)90409-X) 7522972 10.1016/0092-8674(94)90409-x

[45] Bruce DL Macartney T Yong W Shou W Sapkota GP 2012 Protein phosphatase 5 modulates SMAD3 function in the transforming growth factor-β pathway. Cell Signal 24, 1999–2006. (doi:10.1016/j.cellsig.2012.07.003) 22781750 10.1016/j.cellsig.2012.07.003

[46] Niewkoop PD Faber J 1975 Normal Table of *Xenopus laevis* (Daudin). Amsterdam, The Netherlands: North Holland.

[47] Smith JC 1993 Purifying and assaying mesoderm-inducing factors from vertebrate embryos. In Cellular interactions in development—a practical approach (ed. Hartley D), pp. 181–204. Oxford, UK: Oxford University Press.

[48] Slack JM 1984 Regional biosynthetic markers in the early amphibian embryo. J. Embryol. Exp. Morphol. 80, 289–319. 6747529

[49] Vogt J Dingwell KS Herhaus L Gourlay R Macartney T Campbell D Smith JC Sapkota GP 2014 Protein associated with SMAD1 (PAWS1/FAM83G) is a substrate for type I bone morphogenetic protein receptors and modulates bone morphogenetic protein signalling. Open Biol. 4, 130210 (doi:10.1098/rsob.130210) 24554596 10.1098/rsob.130210 PMC3938053

