# Supplementary material for: USP15 targets ALK3/BMPR1A for deubiquitylation to enhance bone morphogenetic protein signalling
Source: Open Biol. 2014 May 21;4(5):140065. doi: 10.1098/rsob.140065 (PMC4042855; doi:10.1098/rsob.140065)
Supplement: Supplementary Figure Legends and Data [file rsob140065supp1.pdf]

SUPPLEMENTARY MATERIAL

USP15 targets ALK3/BMPR1A for deubiquitylation to enhance BMP signalling.

Lina Herhaus[1,4], Mazin A. Al-Salihi[1,3,4], Kevin S. Dingwell[2], Timothy D. Cummins[1], Lize Wasmus[1], Janis Vogt[1], Richard Ewan[1], David Bruce[1], Thomas Macartney[1], Simone Weidlich[1], James C. Smith[2] and Gopal P. Sapkota[1]*

[1] Medical Research Council Protein Phosphorylation and Ubiquitylation Unit, University of Dundee, Dow Street DD1 5EH, Scotland, UK.
[2] Division of Systems Biology, MRC National Institute for Medical Research, The Ridgeway, Mill Hill NW7 1AA, UK.
[3] Present address: School of Medicine, Jordan University of Science and Technology, Irbid 22110, Jordan.
[4] The authors contributed equally to this work
*Address correspondence to: g.sapkota@dundee.ac.uk

Supplementary Figure 1: USP11 interacts with SMAD6 and SMAD7 with similar affinity. HEK293 cells were transiently transfected with vectors encoding FLAG-control, FLAG-SMAD6 or FLAG-SMAD7 with or without HA-USP11. FLAG-IPs or extract inputs (10 µg) were resolved by SDS-PAGE and subjected to immunoblotting with the indicated antibodies.

Supplementary Figure 2: Analysis of USP11 and USP15 expression in mouse tissues. The indicated mouse tissues were homogenised in lysis buffer. 20 µg of protein extract for each tissue was resolved by SDS-PAGE and immunoblotted with the indicated antibodies.

Supplementary Figure 3: USP15-depletion impacts TGFβ-induced transcription of PAI1 in HaCaT cells. HaCaT cells were transfected with *siFOXO4* control or *siRNA* against UPS15. Cells were serum starved for 12 hours and stimulated with 50 pM TGF-β for 4 hours prior to lysis and RNA isolation. RNA was reverse transcribed to cDNA and qRT-PCR was performed for PAI-1, using GAPDH as a loading control. Data are expressed as fold difference over *siFOXO4* control ± SEM; a one-way ANOVA was performed and each group

was compared (****$p<0.0001$; **$p<0.01$). Inset immunoblot shows USP15 levels in *siFOX04* vs. *siUSP15* depleted cell extracts resolved by SDS-PAGE.

Supplementary Figure 4: HA-USP15 overexpression mitigates the *siUSP15-3* mediated inhibition of BMP-induced SMAD1 phosphorylation. HEK293 cells were transiently transfected with *siFOXO4* or *siUSP15-3*. 24 h post *siRNA* transfection, cells were transfected with mammalian expression vectors encoding HA-control or HA-USP15. Cells were then starved overnight and stimulated with or without BMP for 1 h prior to lysis. Extracts were resolved by SDS-PAGE and subjected to immunoblotting with antibodies against USP15, pSMAD1 and total SMAD1.

Supplementary Figure 5: Subcellular localisation of GFP-USP15, FLAG-ALK3 and HA-SMAD6 by immunofluorescence. (A-E) Fixed cell immunofluorescence was performed on U2OS cells transfected with FLAG-ALK3, HA-SMAD6 and/or GFP-USP15 as indicated. Individual and merged images are shown. Individual transfections show that antibodies do not cross-react. Pictures were acquired using a 60x lens.

Supplementary Figure 6: ALK3 turnover is controlled by polyubiquitin-dependent proteasomal degradation. (A) HEK293 cell were transfected with GFP control, GFP-ALK3 or GFP-ALK3 D380A (kinase dead) vectors. Prior to lysis, cells were treated with 10 µM Bortezomib for 3 hours. GFP-IPs and extract inputs were resolved by SDS-PAGE and subjected to immunoblotting analysis using the indicated antibodies. (B) HEK293 transfected with *Xenopus* ALK3 (xALK3-HA) were treated with or without 20 µM cycloheximide for 24 h prior to lysis. Cells were treated with DMSO control, 100 nM Bafilomycin A1 or 10 µM Bortezomib for 3 h prior to lysis. Extracts or HA-IPs were resolved by SDS-PAGE and

subjected to immunoblotting with the indicated antibodies. (C) HEK293 cells transfected with or without human FLAG-ALK3 were treated with DMSO control, 100 nM Bafilomycin A1 or 10 μM Bortezomib for 3 h prior to lysis. Extracts or FLAG-IPs were resolved by SDS-PAGE and subjected to immunoblotting with the indicated antibodies.

Supplementary Figure 7: *siSMAD6* yields reduction in SMAD6 transcripts. HEK293 cells were transiently transfected with *siFoxO4* or *siSMAD6*. Cells were then washed and harvested 48 h later. The expression of SMAD6 transcript was assessed by qRT-PCR. The error bars indicate SD.

Supplementary Figure 8: Loss of *x*USP15 reduces levels of pSMAD1 without affecting the levels of SMAD1. (A) Embryos injected with 80ng of either xUSP15-MO or control-MO were collected at indicated stages. Lysates were resolved by SDS-PAGE and immunoblotted with antibodies against pSMAD1 and total-SMAD1. (B) Quantitation of (A), expressed as a ratio of signal of pSMAD1:total SMAD1. (C) Embryos were injected with 80 ng of either xUSP15-MO or control-MO and then at stage 8.5 were cultured for the indicated times in the presence of cycloheximide. Lysates were immunoblotted simultaneously for total SMAD1 and α−tubulin. The leftmost lane is the 50kDa molecular weight marker. (D) Embryos at the 1 cell stage were injected with mRNAs and morpholinos as indicated (500pg xAlk3-HA mRNA; 500pg, 1ng, 2ng hUSP15 mRNA; 80 ng xUSP15-MO, 80ng Control-MO). Embryos were harvested at stage 11 and HA-IPs and extract inputs were resolved by SDS-PAGE and immunoblotted with the indicated antibodies.

Supplementary Figure 9: Human *siUSP15#3* causes statistically significant reduction in BMP-induced pSMAD1 levels. Western Blot bands representing phospho-SMAD1 and total

SMAD1 from 5 independent experiments using USP15 *siRNA#3* or FoxO4 *siRNA* were quantified using Image J. Data are represented as mean and error bars indicate standard deviation (n=5). Student's t-test was performed and differences with $p<0.001$ were annotated as ***.

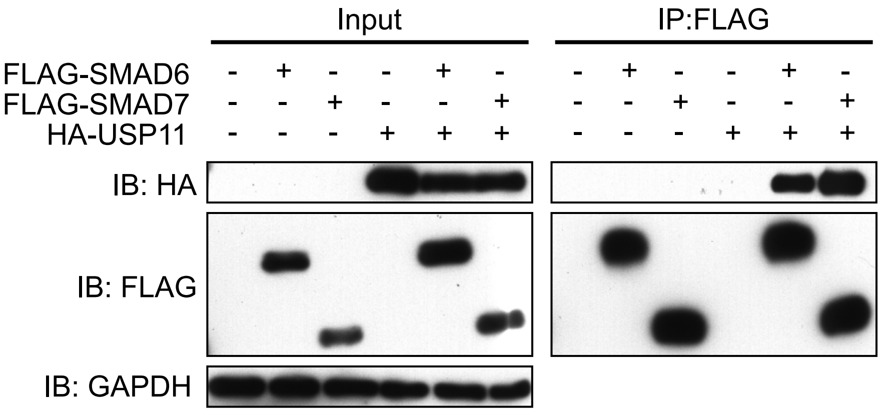

USP15 - Supplementary Figure 1

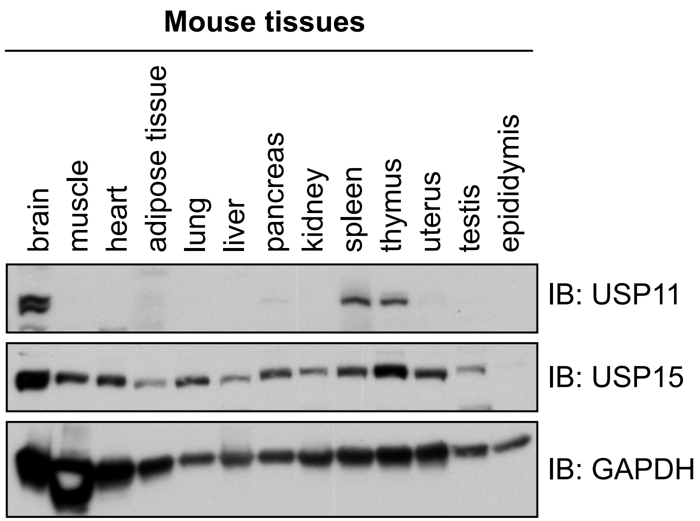

USP15 - Supplementary Figure 2

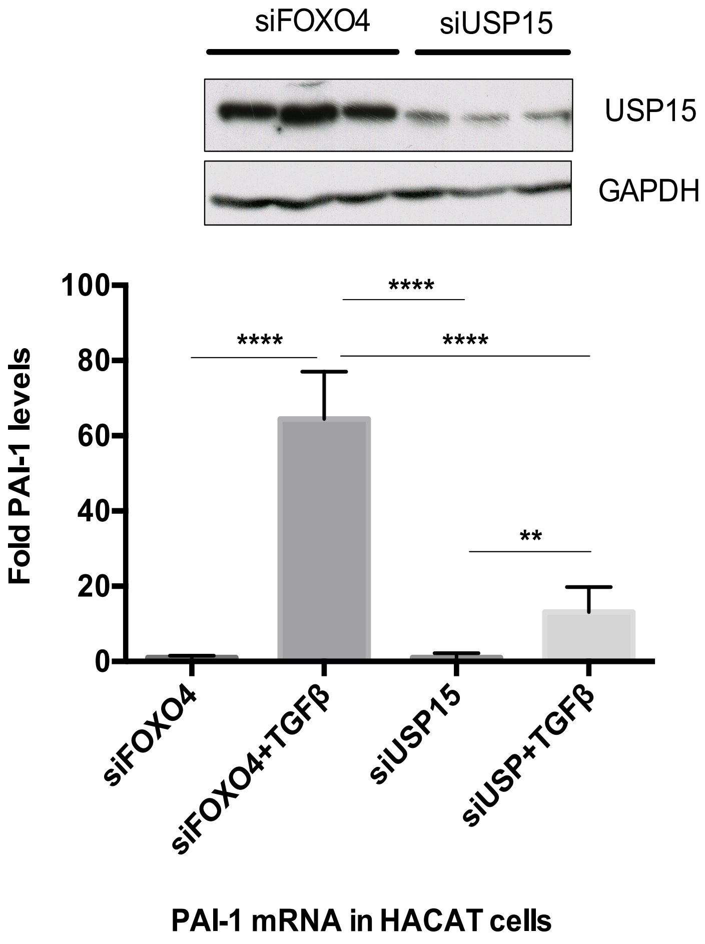

USP15 - Supplementary Figure 3

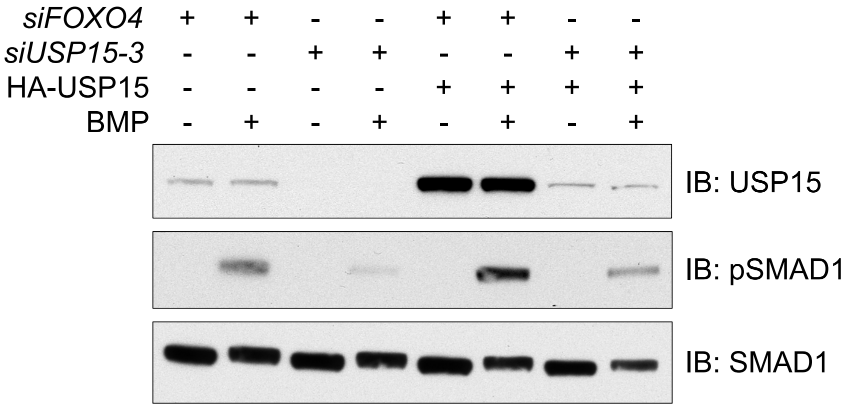

USP15 - Supplementary Figure 4

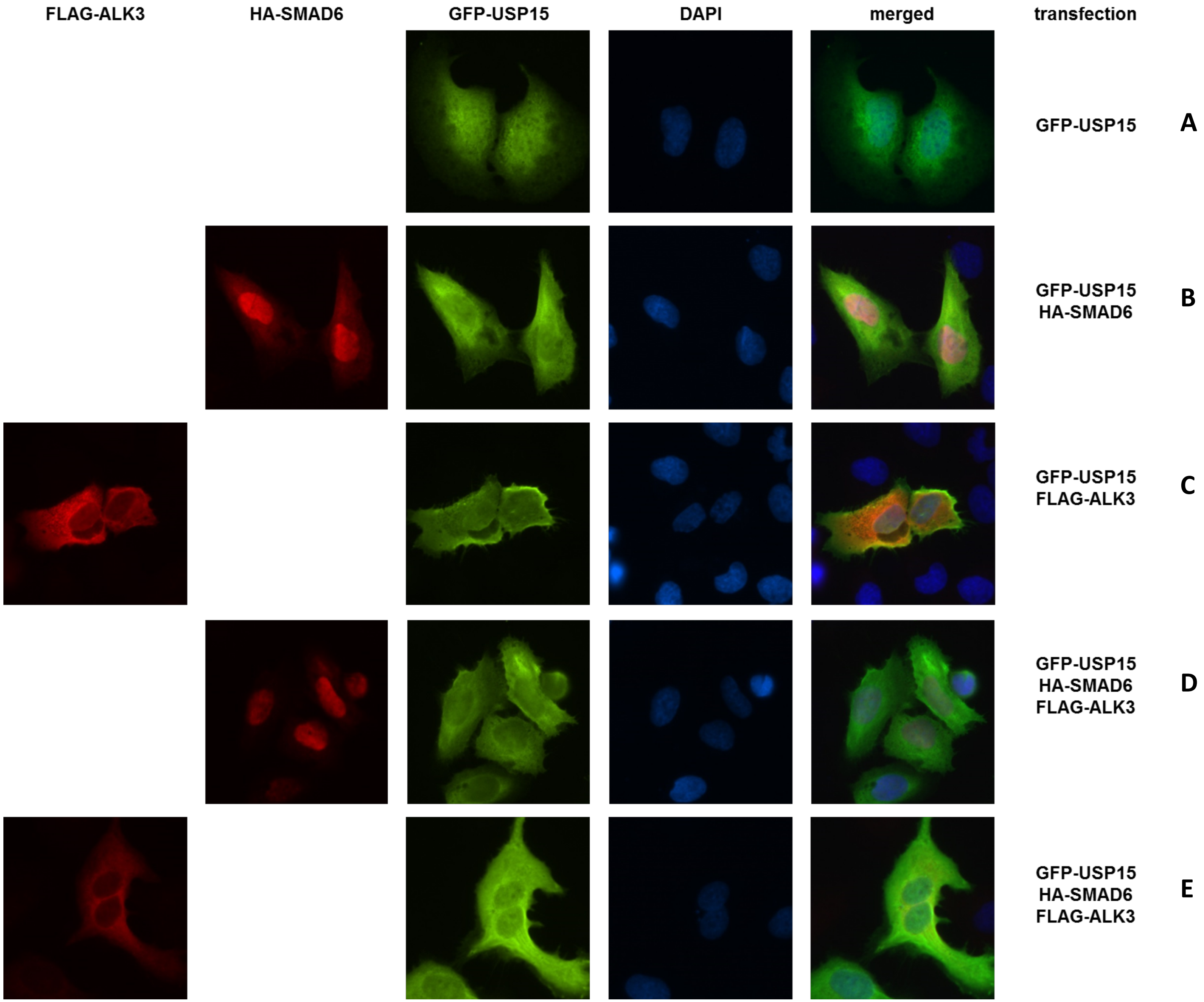

USP15 - Supplementary Figure 5

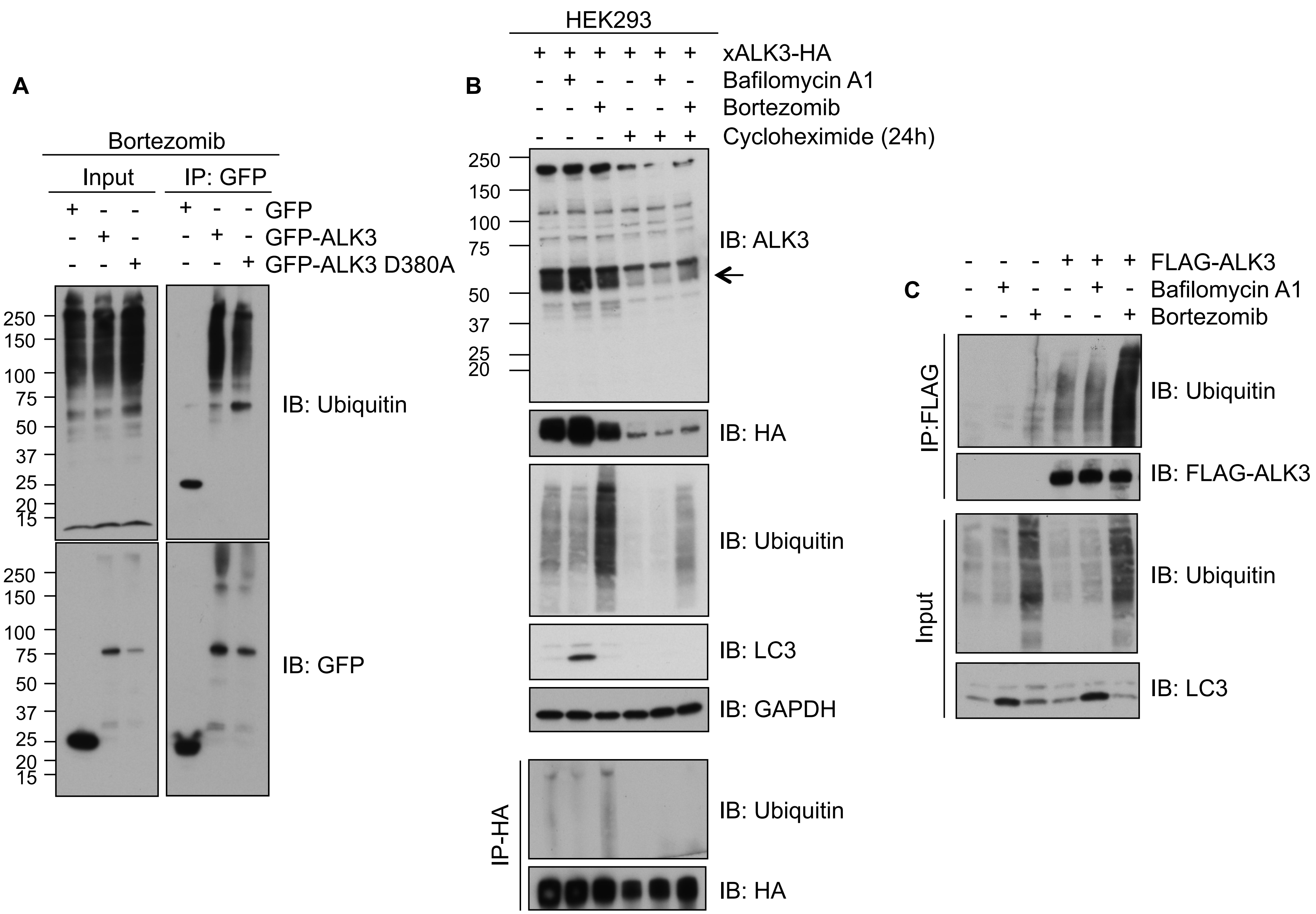
A
Bortezomib
Input
IP: GFP
GFP
GFP-ALK3
GFP-ALK3 D380A
250
150
100
75
50
37
25
20
15
IB: Ubiquitin
IB: GFP
B
HEK293
xALK3-HA
Bafilomycin A1
Bortezomib
Cycloheximide (24h)
IB: ALK3
IB: HA
IB: Ubiquitin
IB: LC3
IB: GAPDH
IP-HA
IB: Ubiquitin
IB: HA
C
FLAG-ALK3
Bafilomycin A1
Bortezomib
IP:FLAG
IB: Ubiquitin
IB: FLAG-ALK3
Input
IB: Ubiquitin
IB: LC3

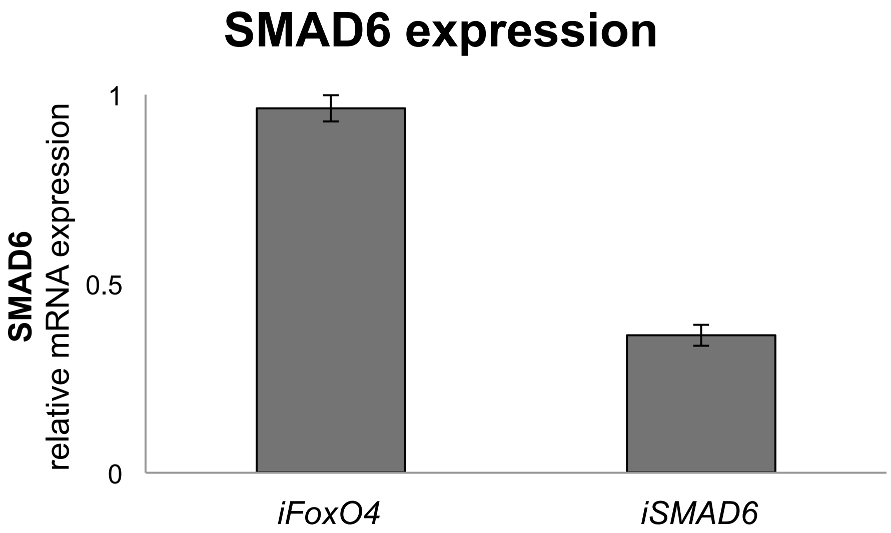

USP15-Supplementary Figure 7

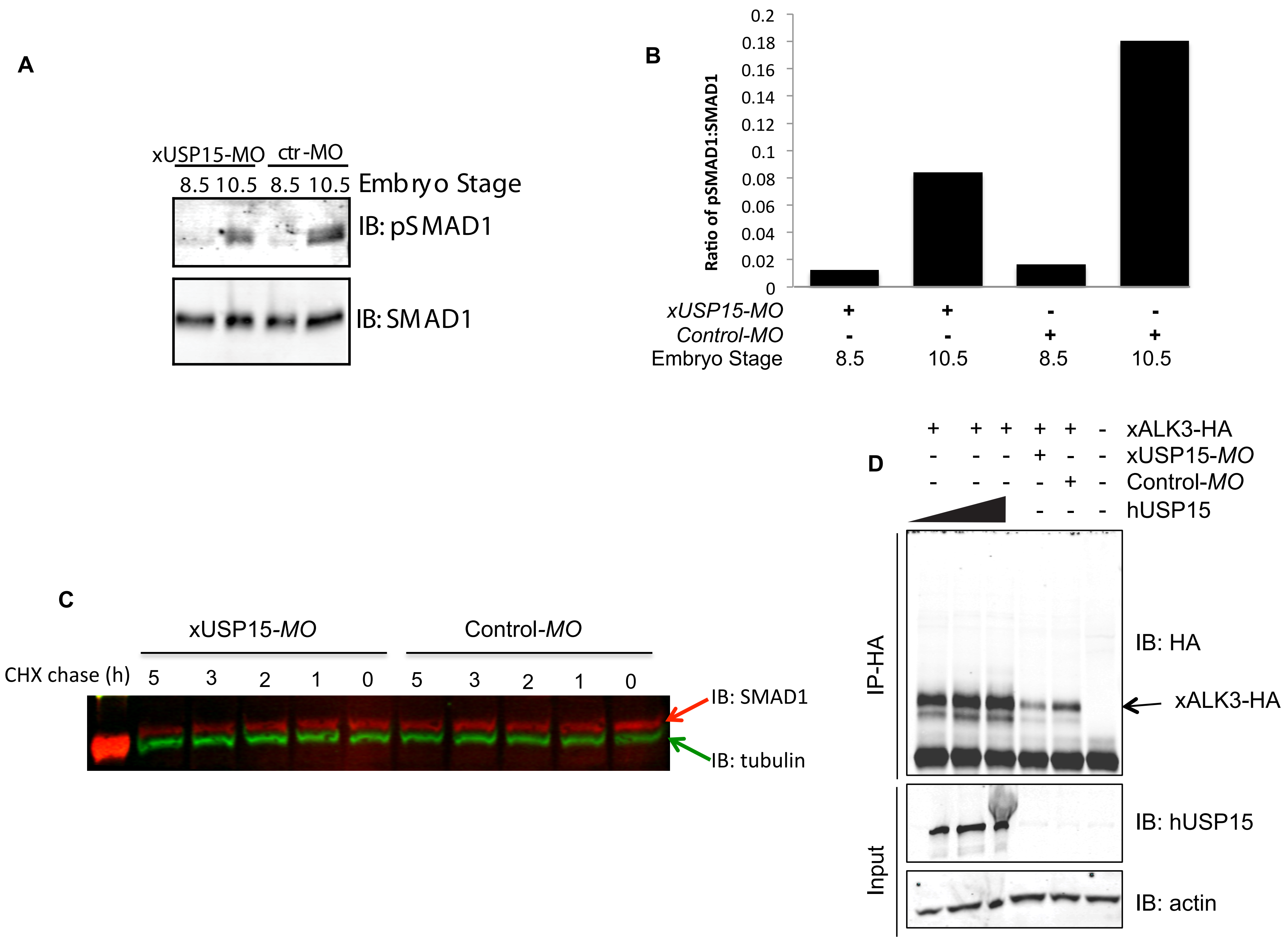

USP15-Supplementary Figure 8

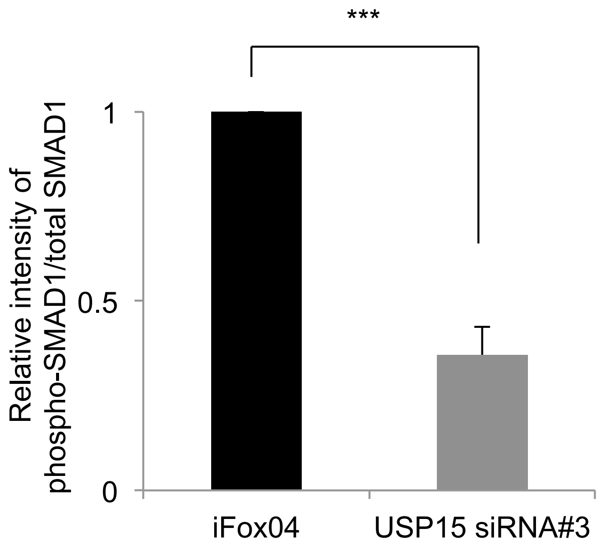

USP15-Supplementary Figure 9
